# Supplementary material for: Migration Characteristics of Manure-Derived Antibiotic-Resistant Bacteria in Vegetables Under Different Soil Types
Source: Microorganisms. 2025 Oct 20;13(10):2398. doi: 10.3390/microorganisms13102398 (PMC12566424; doi:10.3390/microorganisms13102398)
Supplement: Supplementary file 1 [file microorganisms-13-02398-s001.zip › Highlights.pdf]

Highlights

- Pakchoi grown in black soil has the highest abundance of CREB (235.43%)
- CTC in facilitating the spread of opportunistic pathogens
- TN, TP, OM and indigenous antibiotic-resistant bacteria shape CREB communities
- CTC induces the strongest metabolic disruption in pakchoi grown in red soil
