# Supplementary material for: Migration Characteristics of Manure-Derived Antibiotic-Resistant Bacteria in Vegetables Under Different Soil Types
Source: Microorganisms. 2025 Oct 20;13(10):2398. doi: 10.3390/microorganisms13102398 (PMC12566424; doi:10.3390/microorganisms13102398)
Supplement: Supplementary file 1 [file microorganisms-13-02398-s001.zip › supplemental materials.pdf]

Supplementary material for

Migration Characteristics of Manure-Derived Antibiotic-Resistant Bacteria in Vegetables Under Different Soil Types

Tingting Song [1,2], Changxiong Zhu [2,3], Honghui Teng [1], Binxu Li [2], Shuang Zhong [1], Yan Qin [1], Jiawei He [1] and Hongna Li [2,*]

1 College of Engineering, Jilin Normal University, Siping 136000, China; songtingting0505@163.com (T.S.); tenghonghui@163.com (H.T.); ongzhish@126.com (S.Z.); qin.yan.19891017@163.com (Y.Q.); hejiaweispjl@163.com (J.H.)
2 Institute of Environment and Sustainable Development in Agriculture, Chinese Academy of Agricultural Sciences, Beijing 100081, China; zhucx120@163.com (C.Z.); libinxu123@163.com (B.L.)
3 College of Environmental Science and Engineering, Hebei University of Science and Technology, Shijiazhuang 050018, China
* Correspondence: lihongna@caas.cn; Tel.: +86-13811997656

Total number of pages: 4

Number of figures: 2

Number of tables: 1

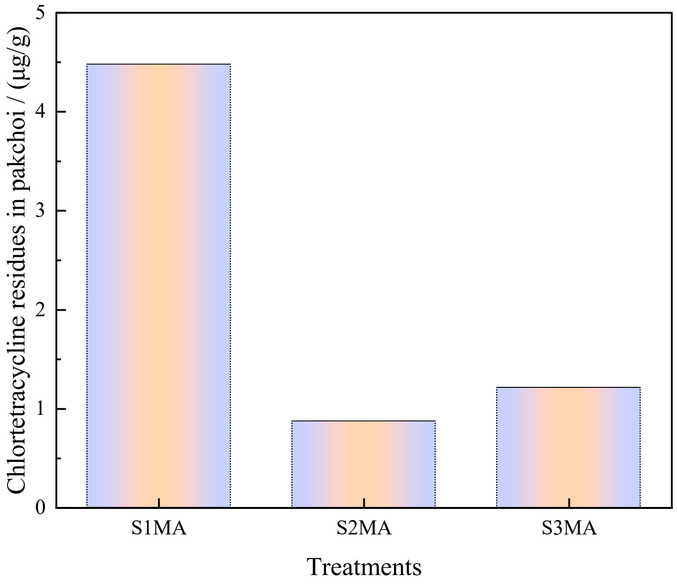

Figure S1. The residual concentration of chlortetracycline in pakchoi

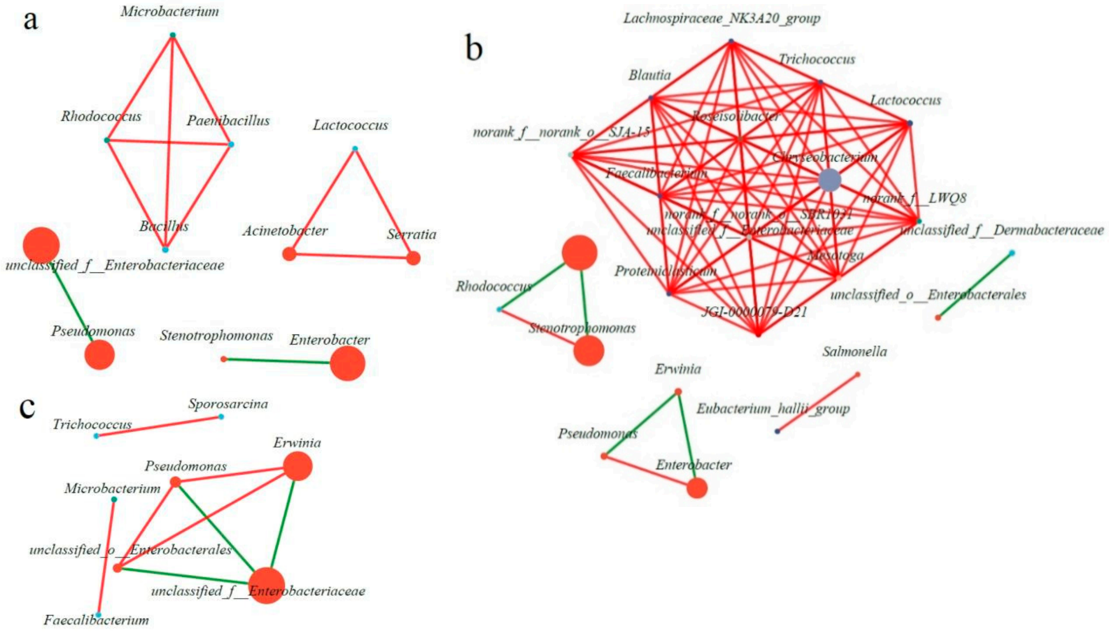

Figure S2. Analysis of single-factor network diagrams among bacterial genera. CREB of pakchoi grown in black soil (**a**). Grown in fluvo-aquic soil (**b**). Grown in red soil (**c**).

Table S1 Changes of physical and chemical properties of different soil types

Treatments	pH	EC (μS/cm)	TN (g/kg)	TP (mg/kg)	OM (g/kg)	CTC (μg/kg)
S1	8.31±0.07a	103.17±9.32a	1.23±0.00a	3.29±0.01b	37.45±0.26a	-
S1M	8.33±0.02a	106.43±3.23a	1.30±0.01a	3.36±0.03a	35.83±0.12c	-
S1MA	8.35±0.03a	112.27±8.15a	1.17±0.12a	3.36±0.00a	36.57±0.37b	24.48±1.67
S2	8.36±0.04a	85.23±5.42b	0.66±0.05a	1.86±0.03b	14.03±0.41a	-
S2M	8.36±0.04a	72.03±6.75c	0.57±0.02ab	1.91±0.02ab	14.05±0.16a	-
S2MA	8.36±0.04a	99.70±7.37a	0.52±0.06a	1.94±0.02a	13.90±0.01a	16.87±0.54
S3	5.67±0.03a	39.43±2.75a	0.62±0.06a	1.46±0.01c	12.86±0.21b	-
S3M	5.58±0.02a	30.80±0.44b	0.63±0.02a	1.57±0.00a	13.91±0.07a	-
S3MA	5.56±0.03a	29.20±2.52b	0.57±0.02a	1.53±0.01b	13.70±0.32a	2.26±0.51

Note: "-" indicates no detection
